# Antibiotic Resistance Pattern of *Staphylococcus Aureus* Isolated From Pediatrics With Ocular Infections: A 6-Year Hospital-Based Study in China

**DOI:** 10.3389/fped.2021.728634

**Published:** 2021-11-19

**Authors:** Xiao-Yu Zheng, Bonnie Nga Kwan Choy, Ming-Ming Zhou, Zheng-Yan Zhao

**Affiliations:** ^1^Department of Ophthalmology, National Clinical Research Center for Child Health, The Children's Hospital, Zhejiang University School of Medicine, Hangzhou, China; ^2^Department of Ophthalmology, Li Ka Shing (LKS) Faculty of Medicine, The University of Hong Kong, Hong Kong, China; ^3^Department of Clinical Lab, National Clinical Research Center for Child Health, The Children's Hospital, Zhejiang University School of Medicine, Hangzhou, China; ^4^Department of Child Health Care, National Clinical Research Center for Child Health, The Children's Hospital, Zhejiang University School of Medicine, Hangzhou, China

**Keywords:** *Staphylococcus aureus*, methicillin-resistant *Staphylococcus aureus*, multidrug resistance, ocular infection, pediatrics, antimicrobial susceptibility

## Abstract

*Staphylococcus aureus* (*S. aureus*) is an important pathogen of ocular infections in pediatrics. The study aimed to identify the prevalence and resistance pattern of *S. aureus*, especially methicillin-resistant *S. aureus* (MRSA), in Chinese children with ocular infections. All patients with *S. aureus* infections were reviewed at a tertiary children's hospital during 2015–2020, and those with ocular infections were investigated for susceptibility results. Of 1,668 *S. aureus* strains, there were 177 unique isolates from ocular infection. Among them, 45 (25.4%) were MRSA and 132 (74.6%) were methicillin-sensitive *S. aureus* (MSSA). The proportion of MRSA did not change over time. Most of the strains were obtained from the neonate ward and ophthalmology department (*n* = 88, 49.7%, and *n* = 85, 48.0%, respectively), while eye secretion and pus were the main specimen types (*n* = 128, 72.3%, and *n* = 37, 20.9%, respectively). MRSA was significantly resistant against penicillin class (97.8%), erythromycin (71.1%), clindamycin (71.1%), and tetracycline (32.1%), with a high multidrug resistance (MDR) rate of 71.1%. However, MRSA was highly sensitive to levofloxacin. Resistance rates against erythromycin and ciprofloxacin as well as MDR percentage all increased among MSSA in children above 1 year of age, ophthalmology department, and outpatient population and decreased in eye secretion specimen. The mean resistance percentage remained stable for MRSA and MSSA during the study period. The survey of ocular *S. aureus* pathogens in pediatrics and their antibiotic resistance patterns helps in clinical treatment. MRSA with many strains demonstrating MDR is highly prevalent in children with ocular infections in Southeast China. Levofloxacin is an effective topical antibiotic for ocular MRSA infection, while erythromycin has a high resistance rate. The antibiotic resistance patterns of MRSA and MSSA differs and varies by different stratifications. A cautious use of antibiotics should be considered.

## Introduction

*Staphylococcus aureus* (*S. aureus*) is a common bacterial pathogen that causes a variety of infections in humans. An increasing number of methicillin-resistant *S. aureus* (MRSA) strains are isolated in hospitals and communities. Being the most significant multidrug-resistant bacteria, MRSA leads to more than 450,000 infections per year (1). The prevalence of MRSA infections in the Asia Pacific area was comparable to those in other regions in the world, and China reported a rate reaching up to 55.3% (2). Besides, a considerably high percentage of MRSA carriage was also found in healthy population in China (3).

It is noteworthy that a diversity of ocular infections caused by MRSA has been reported, such as conjunctivitis, dacryocystitis, orbital cellulitis, keratitis, endophthalmitis, and postoperative infection (4–9). Some of the infections above would harm vision or bring serious complications that threaten life, and this should be a matter of concern (10). Compared to adults, children are more vulnerable to ocular *S. aureus* infection due to vertical transmission, bad hygiene habit, weakened immune systems, and poor compliance to topical eye agents, in addition to the strong infectivity of the pathogen.

Antibiotic selection for children differs from that for adults, because of the distinct antimicrobial-associated adverse events in children. Clinically, the empirical antimicrobial treatments are usually initiated before the culturing and susceptibility results could be obtained. Thus, an up-to-date analysis of antimicrobial susceptibility for local *S. aureus* colonies should be informed to the ophthalmologists and other physicians who deal with pediatric ocular infections, especially for MRSA, which has a high rate of multidrug resistance (MDR).

Antibiotic resistance analyses on pediatric ocular infection due to *S. aureus* in general, and MRSA in particular, are limited. Most of the documentations originated from United States (11–13), and some focused on only one kind of ocular infectious disease (7, 14–16). Few literatures looked into Chinese children, who make up a large part of children worldwide, and some relevant studies with big datasets did not distinguish children from adult population (17, 18). Since susceptibility of MRSA varies by geography and changes over time (19, 20), it is worthwhile to investigate pediatric ocular infection with MRSA in China.

The aim of the study was to evaluate the prevalence of *S. aureus* in ocular infections in a pediatric hospital in southeast China, analyze the susceptibility of the bacteria, and figure out the resistance changes after stratification by methicillin resistance, and adjustment for age, gender, year, season, department, and specimen type, among which MRSA is a particular concern.

## Materials and Methods

### Study Population and Subgroup Division

The Children's Hospital Zhejiang University School of Medicine is the only tertiary pediatric hospital in Zhejiang province, as well as the National Clinical Research Center for Child Health in China. The child patients in our hospital were representative of the patients in southeast China. All pediatrics patients with *S. aureus* infections between January 1, 2015, and December 31, 2020 were retrospectively reviewed, and the ocular specimen tested positive for *S. aureus* were identified. Only “unique” isolates were included for analysis, which indicates the isolates from different patients, or from one patient with different susceptibility results. If the isolates contributed by a single patient showed the same susceptibility results at different times for retesting, they were excluded from our study. Case demographic and microbiological data were retrieved. Subjects were enrolled from both inpatients and outpatients. The isolates from different departments were also analyzed, because ocular infections may not be the primary symptom and the samples could be obtained from patients with different concomitant diseases in departments other than the ophthalmology department. Specimens were collected by non-invasive and invasive means; the former one was a swab of conjunctival sac from patients with conjunctivitis, and the latter could be ocular surgeries, including abscess drainage, lacrimal duct probing, or incision and curettage surgery. Accordingly, the specimens were mainly classified as eye secretion (from conjunctivitis), pus (from cellulitis, orbital abscess, lid abscess, dacryocystitis/lacrimal abscess/lacrimal duct obstruction), and granuloma (from chalazion).

The study population's age ranged from 0 to 18 years and was categorized into two subgroups, including those <1 year (infant) and 1–18 years (older children). We suppose that undeveloped immune defense abilities, vertical transmission or close contact from maternal colonization, early hospitalization, and special disease spectrum make infants have a different type and susceptibility profile of *S. aureus* organism compared to older children (21).

Since many infections have seasonality, we also subdivided the isolates according to the local season in which they were collected, including winter (December–February), spring (March–May), summer (June–August), and autumn (September–November).

The Ethics Committee of Children's Hospital Zhejiang University School of Medicine approved this retrospective study and waived informed consent to participate who received medical treatment at Children's Hospital Zhejiang University School of Medicine (Number: 2021-IRB-090).

### Antibacterial Susceptibility Testing

The clinical specimens were inoculated onto Columbia blood agar (bioMérieux, Marcy-l'Étoile, France, or BIOIVD, Zhengzhou, China) and incubated in 5% CO_2_ for 18–24 h. The colonies were identified using Matrix-Assisted Laser Desorption Ionization Time of Flight Mass Spectrometry (MALDI-TOF MS, Bruker, Billerica, MA, USA). Antimicrobial susceptibility test was performed with the commercialized microdilution method (VITEK COMPACT, bioMérieux, France) using GP67 or P639 cards, and the isolates were divided into susceptible or resistant (intermediate and resistant) categories according to the breakpoints available from Clinical Laboratory Standards Institute (CLSI) guidelines M100-S30.

A total of 19 representative antimicrobial drugs from 15 different antibiotic classes were tested in the clinical laboratories of our hospital for the present study; these included penicillins (oxacillin, penicillin), fluoroquinolones (ciprofloxacin, levofloxacin, moxifloxacin), tetracyclines (tetracycline, tigecycline), lincosamides (clindamycin), cephems (ceftaroline), lipopeptides (daptomycin), macrolides (erythromycin), aminoglycosides (gentamicin), oxazolidinones (linezolid), nitrofurantonins (nitrofurantoin), streptogramins (quinupristin–dalfopristin), ansamycins (rifampin), dihydrofolate reductase inhibitors (trimethoprim–sulfamethoxazole), lipoglycopeptides (teicoplanin), and glycopeptides (vancomycin). *S. aureus* ATCC29213 was used as quality control strain. MRSA is defined as positive by cefoxitin screening test or resistance to oxacillin (by MIC results). Clindamycin susceptibility was assured by both single-agent susceptibility testing and a D-zone test. The panel of sensitivity tests varied with time, and not all antibiotics were tested in each year. MDR was defined as resistance to at least three classes of antibiotics (22).

### Statistical Methods

Statistical analysis was performed using IBM SPSS Statistics (RRID: SCR_019096, version 20, SPSS Inc., Chicago, IL, USA), Microsoft Excel (RRID: SCR_016137, version 2007, Microsoft Inc., Redmond, WA, USA), and GraphPad Prism (RRID: SCR_002798, version 7.04, GraphPad Software Inc., San Diego, CA, USA). Chi-square test or Fisher's exact test was used for comparison between MRSA and MSSA, and analysis of MDR or cumulative resistance rate was performed by patient age, gender, season, department, inpatient, or specimen. *Post-hoc* test was performed to identify the difference in the specific group. The percentages of antibiotic resistance by different groups were evaluated *via* the Kruskal–Wallis ANOVA or Mann–Whitney U test. Because not all the antibiotics were tested during the study period, we used the means of the percentage of antibiotics to which the isolate was resistant. The linear regression model or linear-by-linear test was applied to demonstrate the changes in resistance or MDR percentage over time. The value of *p* < 0.05 was considered statistically significant.

## Results

### Characteristics of *S. aureuas* Isolates

**Figure 1:**
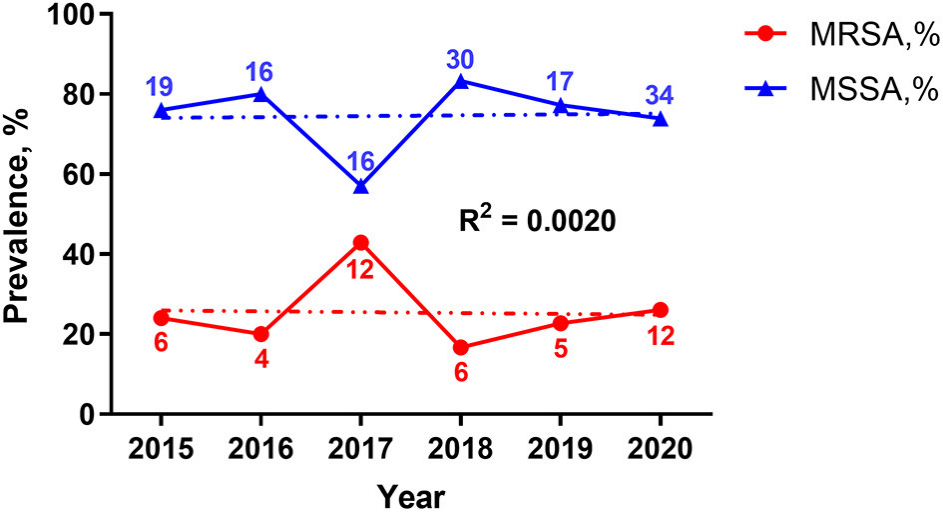
Year-wise prevalence percentage of *S. aureus* isolates during 2015–2020. MRSA and MSSA are shown in red and blue, respectively. The lines represent the percentage of the two isolates per year. The numbers near the lines indicate the actual number of isolates in that year. Trend lines are shown as the dotted ones, with the correlation coefficient *R*^2^ = 0.0020. MRSA, methicillin-resistant *S. aureus*; MSSA, methicillin-sensitive *S. aureus*.

A total of 1,668 *S. aureus* strains were isolated from 1,657 patients with various infections between January 2015 and December 2020 (6 years), among which 191 (11.5%) were obtained from patients with ocular infections. There were 177 unique *S. aureus* isolates obtained from 176 patients. These included 45 (25.4%) MRSA and 132 (74.6%) MSSA. There were 102 (57.6%) male and 75 (42.4%) female. One hundred and twenty-nine (72.9%) isolates were obtained from patients <1 year old, and 48 (27.1%) isolates from those 1–18 years old. Three departments contributed the ocular isolates (88 isolates from the neonate ward; 85 isolates from the ophthalmology department; 4 isolates from other departments including rheumatology and immunology wards, infection ward, hematology clinic, and diabetes clinic). Three kinds of specimens were obtained (128 isolates, eye secretion from conjunctival sac; 37 isolates, pus from palpebral abscess, orbital abscess, and lacrimal abscess; 12 isolates, granuloma from chalazion). Table 1 shows the demographic characteristics among MRSA and MSSA isolates, and a high proportion of MRSA collected from other departments was notable when compared with the neonate or ophthalmology department (*p* = 0.005, Cramer's V = 0.265, adjusted standardized residuals = 3.5; Table 1). No granuloma specimen was cultured positive for MRSA. The prevalence of *S. aureus* strains based on methicillin resistance over time is presented in Figure 1. The total number of *S. aureus* increased during the 6-year study period, but the proportion of MRSA remained relatively stable, despite of a peak in 2017.

**Table 1:** Characteristics of the study population and *S. aureus* isolates.

**Clinical characteristics**	**MRSA (*****n*** **=** **45)**		**MSSA (*****n*** **=** **132)**		**χ^2^**	***p* ^**a**^**
	**No. of isolates**	**%**	**No. of isolates**	**%**		
**Age**					0.2185	0.640
<1 year	34	75.6	95	72.0		
1–18 years	11	24.4	37	28.0		
**Gender**					0.139	0.709
Male	27	60.0	75	56.8		
Female	18	40.0	57	43.2		
**Season**					6.157	0.104
Winter	16	35.6	30	22.7		
Spring	9	20.0	21	15.9		
Summer	8	17.8	48	36.4		
Autumn	12	26.7	33	25.0		
**Department**						0.005*
Neonate	19	42.2	69	52.3		
Ophthalmology	22	48.9	63	47.7		
Others	4	8.9	0	0		
**Inpatient**					0.003	0.958
Inpatient	24	53.3	71	53.8		
Outpatient	21	46.7	61	46.2		
**Specimen**					5.0587	0.080
Eye secretion	33	73.3	95	72.0		
Pus	12	26.7	25	18.9		
Chalazion granuloma	0	0	12	9.1		

a Fisher's exact test for department, chi-square test for the others. *p < 0.05.

*MRSA, methicillin-resistant S. aureus; MSSA, methicillin-sensitive S. aureus*.

### Resistance Analysis

Table 2 represents the resistance profiles for MRSA and MSSA. Among MRSA isolates, cumulative resistance was high for penicillins class (penicillin and oxacillin, 97.8%, respectively), erythromycin (71.1%), clindamycin (71.1%), and tetracycline (32.1%), and only a small proportion were resistant to nitrofurantoin (9.1%), ciprofloxacin (7.1%), trimethoprim–sulfamethoxazole (4.4%), gentamicin (2.2%), rifampin (2.2%), and fluoroquinolones class (levofloxacin and moxifloxacin, 2.8%, respectively). All MRSA isolates were susceptible to tigecycline, ceftaroline, daptomycin, linezolid, quinupristin-dalfopristin, teicoplanin, and vancomycin. With the exception of no isolates found resistant to oxacillin, rifampin, and nitrofurantoin, MSSA exhibited lower resistance rates of 50, 49.2, and 14.1% to erythromycin, clindamycin, and tetracycline, respectively, compared to MRSA (*p* < 0.050).

**Table 2:** Antimicrobial susceptibility testing results among MRSA and MSSA.

**Antimicrobial drugs**	**MRSA**			**MSSA**			**OR (CI)**	***p* ^**a**^**
	**No. of isolates**	**No. of resistant isolates (%)**		**No. of isolates**	**No. of resistant isolates (%)**			
Oxacillin	45	44	(97.8)	132	0	(0)	133.0 (18.9–937.2)	<0.001*
Penicillin	45	44	(97.8)	132	116	(87.9)		0.098
Ciprofloxacin	28	2	(7.1)	85	6	(7.1)		1.000
Levofloxacin	45	1	(2.2)	132	8	(6.1)		0.536
Moxifloxacin	45	1	(2.2)	132	8	(6.1)		0.536
Tetracycline	28	9	(32.1)	85	12	(14.1)	2.88 (1.06–7.84)	0.033*
Tigecycline	45	0	(0)	132	0	(0)		
Clindamycin	45	32	(71.1)	132	65	(49.2)	2.54 (1.22–5.26)	0.011*
Ceftaroline	13	0	(0)	41	0	(0)		
Daptomycin	3	0	(0)	6	0	(0)		
Erythromycin	45	32	(71.1)	132	66	(50.0)	2.46 (1.19–5.11)	0.014*
Gentamicin	45	1	(2.2)	131	1	(0.8)		0.447
Linezolid	45	0	(0)	132	0	(0)		
Nitrofurantoin	11	1	(9.1)	33	0	(0)		0.250
Quinupristin–dalfopristin	28	0	(0)	85	0	(0)		
Rifampin	45	1	(2.2)	132	0	(0)		0.254
Trimethoprim–sulfamethoxazole	45	2	(4.4)	132	9	(6.8)		0.832
Teicoplanin	16	0	(0)	47	0	(0)		
Vancomycin	45	0	(0)	132	0	(0)		

a Fisher's exact test for gentamicin, nitrofurantoin, and rifampin, chi-square test for the others. *p < 0.05.

*MRSA, methicillin-resistant S. aureus; MSSA, methicillin-sensitive S. aureus; OR (CI), odd ratio (confidence interval)*.

**Figure 2:**
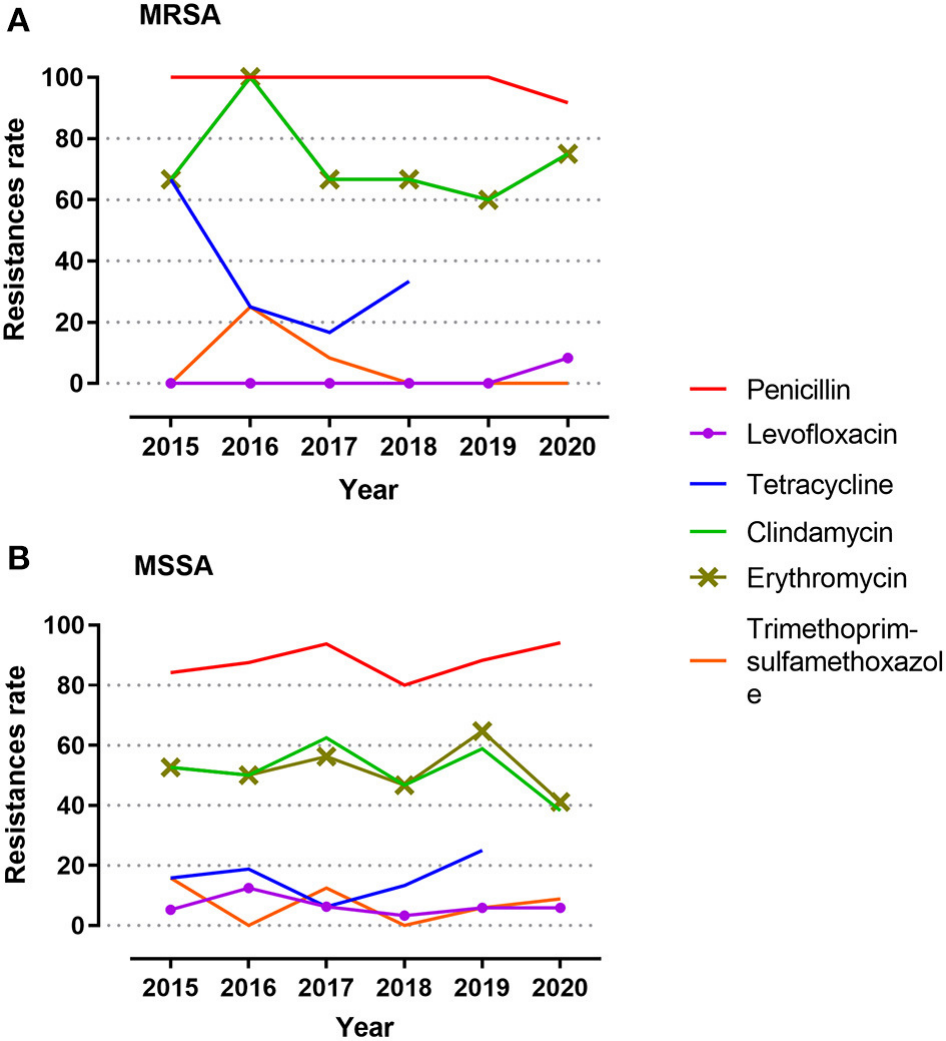
Antibiotic resistance during the 6-year study period. **(A)** Resistance rates among MRSA. **(B)** Resistance rates among MSSA. Isolates were tested against penicillin, levofloxacin, tetracycline, clindamycin, erythromycin, and trimethoprim–sulfamethoxazole. Reasons for exclusion of antibiotics herein includes same antimicrobial drug class, 100% susceptibility, or few isolates tested during the study year. MRSA, methicillin-resistant *S. aureus*; MSSA, methicillin-sensitive *S. aureus*.

**Figure 3:**
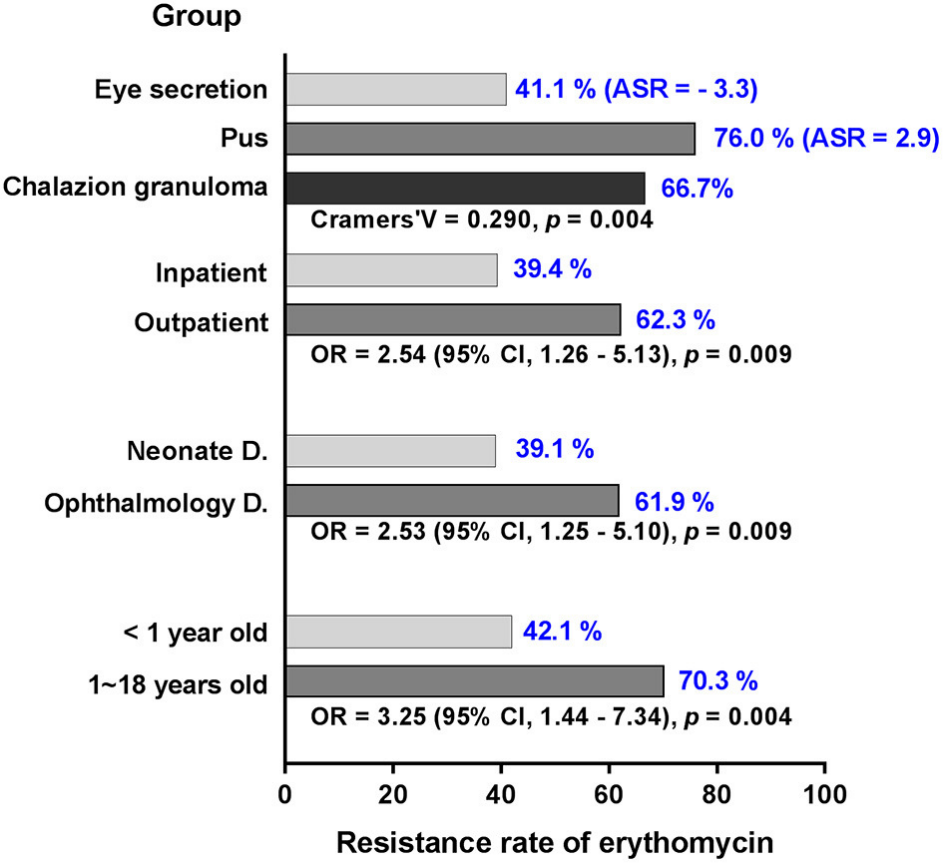
Significant variance of MSSA in resistance rate against erythromycin in different settings. The resistance rate (blue numbers) of MSSA is stratified by groups of age, department, inpatient, and specimen. With the exception of adjusted standardized residuals applied for specimen, odd ratio and confidence interval are used to demonstrate significant differences in the groups. MSSA, methicillin-sensitive *S. aureus*; D., department; ASR, adjusted standardized residuals; OR, odd ratio; CI, confidence interval.

For each of the antibiotics, the resistance rate did not change significantly over time (Figure 2). MRSA showed no difference in resistance rate between groups; however, MSSA demonstrated some variances for erythromycin and clindamycin. MSSA strains isolated from children above 1 year of age, ophthalmology department, and outpatient population all presented high resistance rates against erythromycin and clindamycin, and the strains obtained from eye secretion specimen showed a decreased rate (*p* < 0.050; Figure 3). Because erythromycin and clindamycin shared similar resistance characteristics by groups, only the resistances of erythromycin are plotted in Figure 3.

### MDR

**Figure 4:**
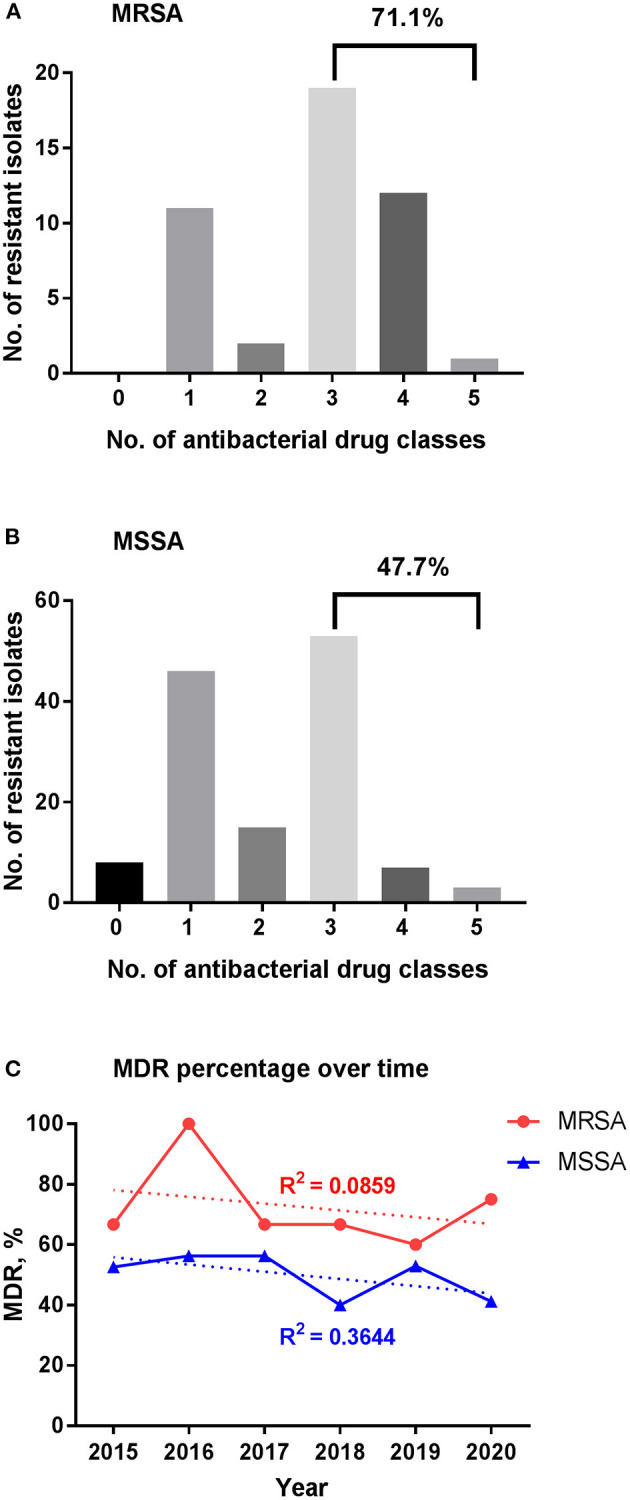
Multidrug resistance among *S. aureus* isolates. **(A)** MDR for MRSA. **(B)** MDR for MSSA. **(C)** Change of MDR percentage over time. The percentage of MDR for MRSA and MSSA isolates stratified per year are shown in red and blue, respectively; trend lines are shown as the dotted ones, with the correlation coefficient *R*^2^ = 0.0859 and 0.3644 for MRSA and MSSA, respectively. MDR, multidrug resistance; MRSA, methicillin-resistant *S. aureus*; MSSA, methicillin-sensitive *S. aureus*.

MDR *in vitro* was found in 71.1% of MRSA and 47.7% of MSSA isolates (Figure 4). The mean percentage of MDR was 72.5% for MRSA during the 6-year study period with a peak of 100% in 2016, while few changes were seen in MSSA stains over time with a mean percentage of 49.9%.

**Figure 5:**
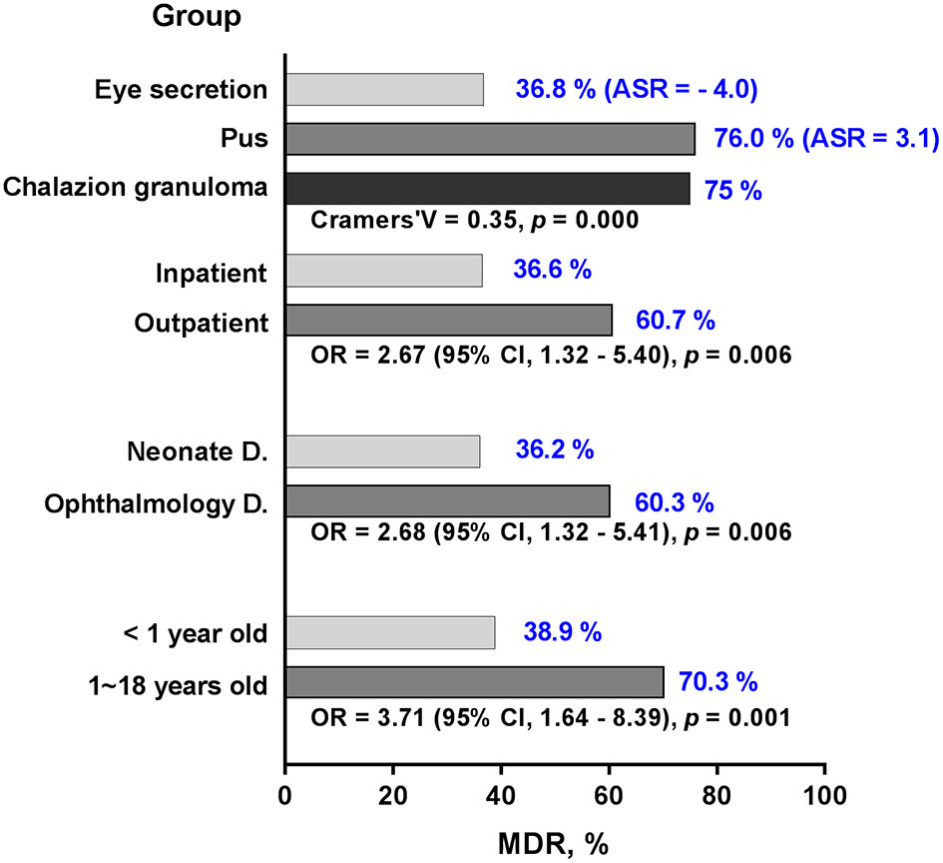
Multidrug resistance percentage of MSSA by different groups. The percentage of MDR (blue numbers) among MSSA are stratified by groups of age, department, inpatient and specimen. With the exception of adjusted standardized residuals applied for specimen, odd ratio and confidence interval are used to demonstrate significant differences in the groups. MDR, multidrug resistance; MSSA, methicillin-sensitive *S. aureus*; D., department; ASR, adjusted standardized residuals; OR, odd ratio; CI, confidence interval.

The percentage of MDR among MSSA was higher in children older than 1 year compared to younger patients (*p* = 0.001; Figure 5). Ophthalmology department and outpatient group had more MDR of about 60% (*p* = 0.006, respectively). The percentage of MDR also differed by specimen group, with the highest rate observed in pus and lowest rate in eye secretion (*p* < 0.001).

### Mean Percentage of Antibiotic Resistance

**Figure 6:**
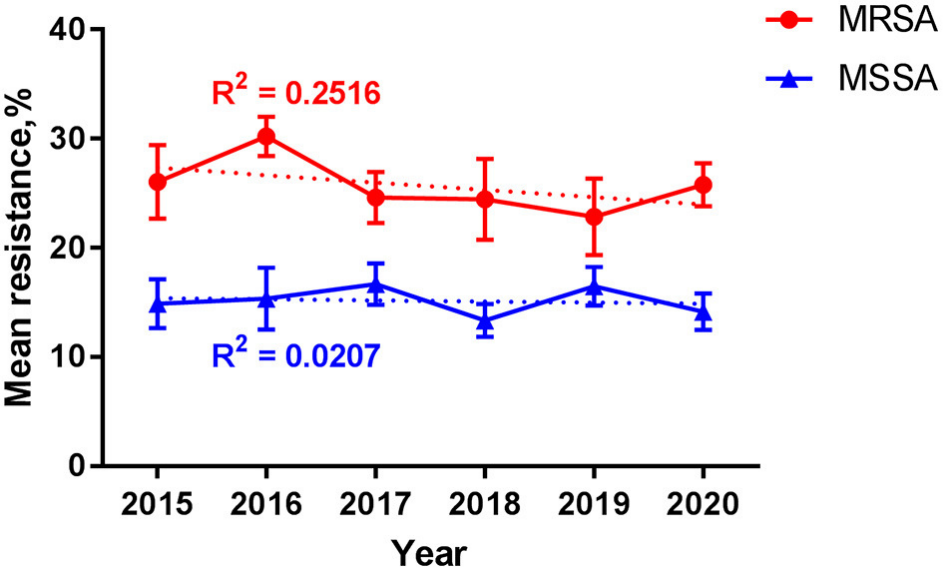
Resistance among *S. aureus* over time. Data are presented as mean (standard error) percentage of resistance for MRSA (red line) and MSSA (blue line) by year. Trend lines are shown as the dotted ones, with the correlation coefficient *R*^2^ = 0.2516 and 0.0207 for MRSA and MSSA, respectively. MRSA, methicillin-resistant *S. aureus*; MSSA, methicillin-sensitive *S. aureus*.

Over the 6 years, the mean (SE) resistance percentage of MRSA ranged from 22.9 (3.5)% to 30.2 (1.8)% and that of MSSA from 13.3 (1.6)% to 16.7 (1.9)% (Figure 6). Among MRSA and MSSA isolates, no changes were found in the mean resistance percentage over year during the study period (*p* = 0.624 and 0.175, respectively), or by different groups such as gender, season, department, inpatient and specimen. A slight but insignificant increase in the mean resistance rate was detected for MRSA in patients beyond 1 year of age compared with their counterpart under 1 (29.1 vs. 24.2%, *p* = 0.051).

## Discussion

Our study was unique for it was an updated 6-year survey of ocular *S. aureus* involving a large population representative of the pediatrics in southeast China. The information of local microbial flora susceptibility usually comes from regional hospitals which contribute a large amount of isolates of both inpatients and outpatients (23), but we are not aware of many studies in China to date. The present findings would help pediatricians in treatment strategies.

Our study revealed that about 11.5% of isolates contributed by pediatrics with *S. aureus* infections were related to the eyes, and 25.4% of ocular *S. aureus* isolates were methicillin resistant (MRSA). The prevalence of MRSA was consistent with the corresponding percentage from the ARMOR study (24.3%, 2009–2016) (13), and much lower than that from an Iranian research (78.6%, 2011–2013) (12). However, there have been few literatures focusing on pediatrics. In the studies involving a broader age range (from infant to elderly), the percentage of MRSA varies a lot: the rates were high in the Ocular TRUST3 study (48.1%, 2007–2008) and the latest ARMOR study (34.9%, 2009–2018) (20, 24), both of which were conducted in United States; the rates were extremely low in Canada (6.4%, 2000–2010) and Polish (6.0%, 2009–2013) (25, 26); and for surveys in Asia, a similar rate of 21% was demonstrated in India (2007–2017) (27), a higher rate of 56.8% in Shanghai (6 year study, uncertain) and 52.8% in Taiwan (1999–2008) (17, 28), and a lower rate of 12.5% In Hong Kong (2005–2015) (18). In general, the rates of *S. aureus* cultured positive for MRSA over the world were variable depending on geography and year of study, and most of the rates were relatively high throughout the past two decades; more attention should be paid to it.

In the current study, the *S. aureus* strains were most frequently isolated from departments of neonate and ophthalmology. Only four isolates were contributed by other departments, and they were all cultured positive for MRSA. We presume there could be two reasons for the high rate of MRSA in other departments: (1) the patients tended to have concurrent immune disorders or mixed infections, and (2) the physicians might not pay attention to ocular infections unless they appeared serious or refractory, which was suggested by the low bacteria examination rate. The present findings should remind the physicians from departments other than ophthalmology to stress the importance of ophthalmic culturing when the patients present symptoms of ocular infections.

In the specimens cultured positive for *S. aureus*, eye secretion from the conjunctival sac represented the majority, of which 25.8% was proven to be MRSA. The proportion was higher than that from the ARMOR study, which reported a rate no more than 15% in the patients under 20 years of age (2009–2013) (29), and lower than 40% from the study of Malena (2002–2009) (11). Ahmad reported that MRSA species was detected in 3 of 12 children with conjunctivitis aging from neonate to 7 years, but the participants were not representative of all ages of children and the sample size was small (14). Neonatal conjunctivitis is prevalent; when we categorized the isolates collected from eye secretions according to patient age, 87.5% were obtained from children under 1 year of age. However, no difference of MRSA proportion was found in children under 1 year of age compared to those beyond, and MRSA colonized in conjunctival sac did not seem to cause severe ophthalmic infection to the newborn (4). Even so, conjunctival MRSA infection should not be underestimated in infants because the virulence of MRSA changes (26, 30), and infants are extremely susceptible to bacterial infection due to their pre-matured immune system.

In the present study, we included two kinds of specimen which were obtained *via* surgery, pus, and granuloma. Pus was mainly isolated from patients with cellulitis, orbital abscess, lid abscess, dacryocystitis, or lacrimal duct obstruction. Some studies reviewed similar diseases, but they did not describe the type of specimen and the method of sample collection (18, 31). In the study by Nithyaa, the pus was collected using a conjunctival swab in patients with pre-septal cellulitis (27). Most of the previous studies subdivided the isolates by clinical entities, but we believe the classification of specimens would give some other information, because the pus drained surgically is relatively deep in tissue, whose bacterium is supposed to have different characteristics compared to that from the superficial specimen. Our findings showed that 32.4% of pus specimens were methicillin resistant, and the rate was higher than that of eye secretion. For the other type of surgical specimen, granuloma from chalazion was reported to be cultured positive for *S. aureus* (32); however, no MRSA was isolated in our study.

There were numerous studies suggesting the seasonality of *S. aureus* infection, and warmer seasons are believed to correlate with increased MRSA colonization, with the peak in late summer or early autumn (33, 34). Most of the studies investigated infections in skin and soft tissue, and few focused on ocular infections. However, our study did not find any seasonal variations in either the proportion or the resistance patterns of ocular MRSA.

MRSA presented a high rate of concurrent resistance to other commonly used antibiotic classes, with 71.1% strains demonstrating MDR. To be specific, MRSA isolates were two to three times more likely to be resistant to tetracycline, clindamycin, and erythromycin when compared with MSSA. The ARMOR study showed that MRSA had a low resistance against clindamycin (4.6%), and MSSA even lower. However, we saw a really high corresponding rate in the present analysis (71.1%). For ciprofloxacin, the situation was opposite; our study was different from ARMOR in showing good susceptibility to ciprofloxacin (13). This might be related to the frequent use of antibiotics; for instance, we do not use topical ciprofloxacin routinely for ocular infection, but erythromycin is commonly used in China. Interestingly, the ARMOR study also noted a small but significant decrease in the percentage of isolates resistant to ciprofloxacin among *S. aureus* (13), which possibly implies fewer clinical application of this agent in recent years.

For MRSA, the present study detected no changes over time with respect to its proportion in *S. aureus* isolates or its resistance against variable antibiotics. The trend of MRSA prevalence remained stable in the 6-year period, which was consistent with the study of Chuang (17), although a study from Japan reported a decline from 52 to 22% in two periods of time with a 10-year interval (35). A small decrease in MRSA prevalence was seen in ARMOR reporting from US (2009–2018), while a steady increase from 9 to 38% was found in India during almost the same period of time (2007–2017) (27), and also an increase from 0.5 to 12.6% in Canada in an earlier period (2000–2010) (25). Stratification by different groups did not report any difference for the MRSA isolate, except for a small increase in the mean percentage of resistance in the children beyond 1 year of age. The previous studies demonstrated that the resistance rate was higher in elderly patients (20, 36), but in the pediatric period no variance of resistance rate was reported (13). Presumably, the resistance among MRSA has a tendency to increase with age, but the variance is small in childhood.

For MSSA, we found some significant differences in the percentage of MDR as well as the resistance rate against erythromycin and clindamycin (due to inducible resistance of erythromycin to clindamycin, we only discussed erythromycin hereinafter). They differed in almost the same groups with similar pattern; to be specific, MDR rate and erythromycin resistance rate both elevated in patients beyond 1 year of age, in the ophthalmology department, and in outpatients compared to their counterparts within the subgroups by patient age and source, whereas both rates decreased in the isolates from eye secretions compared to other types of specimens. In the ophthalmology department, 39.7% of specimens were pus, and 41.3% were eye secretion, but in the neonate department, all the specimens were eye secretion. The specimen distribution pattern was similar in the outpatient group. Most of the eye secretions were obtained from patients with conjunctivitis. Our analysis suggested that, compared to cellulitis, MSSA-related conjunctivitis might easily be cured with common antibiotic eye drugs such as erythromycin, and there was less MDR. In addition, we noticed a rise of erythromycin resistance by patient's age. Topical erythromycin is the most popular over-the-counter antimicrobial drug in the market of China, not only for conjunctivitis but also for bacterial dermatitis, both of which are common diseases in children. Increased antimicrobial resistance would be attributed to abuse of antibiotics without prescription, improper dosage or course of treatment, and misuse of antibiotics in non-bacterial diseases (37).

Sensitivity to fluoroquinolones differs by the agent generations and the countries (26, 38); our study showed extremely low resistance against ciprofloxacin and levofloxacin among MRSA (7.1, 2.8%), much lower than that from the pediatric ARMOR study (40.6–42.0% for fluoroquinolones) (13), and lower than that from the study of Hong in Shanghai, China, which was not limited to children (60.3, 68.6%) (28). Levofloxacin, one of the most commonly used eye drops for pediatrics in China, was found to be more sensitive to ocular MRSA infections than in the United States. An increase in the resistance of ciprofloxacin and levofloxacin with age might be one of the reasons for the difference between pediatrics and the subjects with a broader age range in China. We recommend that prescription of quinolones such as levofloxacin should be cautious to avoid development of resistance from indiscriminate use (23).

The present study is subject to some limitations: (1) certain antimicrobials, for instance tobramycin and chloramphenicol being common topical eye agents, were not routinely tested in our hospital; (2) the specimens collected herein were not multifarious enough, and no corneal scraping from keratitis, humor aqueous from uveitis or endophthalmitis, or tissue samples from infectious ocular surgery were cultured in our hospital during the study period; (3) due to the complicated medication especially in the neonate ward, we did not analyze the number of patients who had received empirical treatment of ocular infection before the culturing and susceptibility results could be obtained; and (4) there were missing parts of medical records for the outpatients, and the isolates were not distinguished between healthcare associated-MRSA (HA-MRSA) and community associated-MRSA (CA-MRSA). However, they might differ in distribution as well as antibiotic susceptibility patterns in pediatrics. In future studies, this would be taken into account because the stratification of HA-MRSA and CA-MRSA is important for the management of MRSA as it remains an important pathogen causing nosocomial and community infections in the region.

In conclusion, MRSA colonization is prevalent with a high rate of MDR in pediatric ocular infection in Southeast China. Culturing and susceptibility tests before initiation of therapy for ocular infections are worthwhile, especially for the physicians in departments other than the ophthalmology department. Resistance of erythromycin is remarkable among MRSA, and there is a tendency to increase with age among MSSA. Topical levofloxacin continues to provide a low level of resistance with broad-spectrum coverage for empirical treatment of ocular infections in children. However, physicians should use it with caution to prevent further development of resistance. In management of ocular infection with MRSA and MSSA, their differences in prevalence and resistance by patient age and specimen source should be carefully considered.

## Data Availability Statement

The original contributions presented in the study are included in the article/Supplementary Material, further inquiries can be directed to the corresponding author.

## Ethics Statement

The studies involving human participants were reviewed and approved by the Ethics Committee of Children's Hospital Zhejiang University School of Medicine. Written informed consent from the participants' legal guardian/next of kin was not required to participate in this study in accordance with the national legislation and the institutional requirements.

## Author Contributions

X-YZ developed the project and wrote the manuscript. BC analyzed the data and revised the manuscript. M-MZ collected the data. Z-YZ developed the project and interpreted the data. All authors read and approved the final manuscript.

## Funding

This study was supported by the Zhejiang Provincial Natural Science Foundation (No. LSY19H180011). The sponsor or funding organizations had no role in the design or conduct of this research.

## Conflict of Interest

The authors declare that the research was conducted in the absence of any commercial or financial relationships that could be construed as a potential conflict of interest.

## Publisher's Note

All claims expressed in this article are solely those of the authors and do not necessarily represent those of their affiliated organizations, or those of the publisher, the editors and the reviewers. Any product that may be evaluated in this article, or claim that may be made by its manufacturer, is not guaranteed or endorsed by the publisher.
